# An exploration of microbial response to stressors with Prof. Claudio C. Vásquez Guzmán

**DOI:** 10.1186/s40659-022-00393-3

**Published:** 2022-08-06

**Authors:** Michael Seeger, Raymond J. Turner, Mauricio González

**Affiliations:** 1 Laboratorio de Microbiología Molecular y Biotecnología Ambiental, Departamento de Química & Centro de Biotecnología Dr. Daniel Alkalay Lowitt, Universidad Técnica Federico Santa María, Avenida España 1680, Valparaiso, Chile; 2 Department of Biological Sciences, University of Calgary, Calgary, AB Canada; 3 Laboratorio de Bioinformática y Expresión Génica, INTA, Universidad de Chile, Santiago, Chile


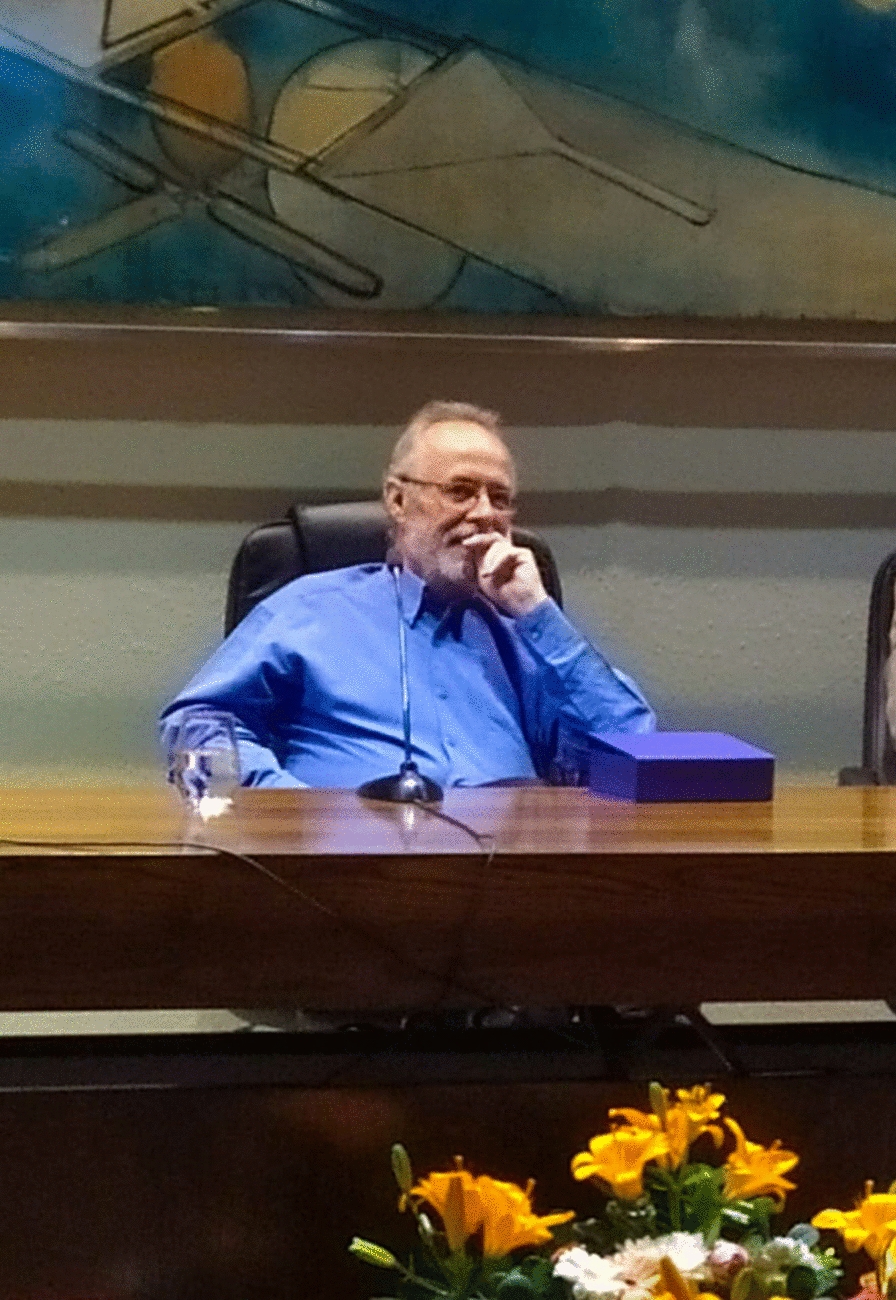
Microorganisms, particularly bacteria, are the major species on the planet, considered to be up to 98% of all species. Bacteria have evolved impressive responses to various stressors, which have been essential for the adaptation and evolution of microorganisms and colonization of a wide range of environments. This special issue of *Biological Research* on microbial response to stressors is a tribute of the Chilean and international scientific community to the late Professor Claudio C. Vásquez Guzmán (1952–2020) who dedicated his career in environmental microbiology and biochemistry to the study of bacterial stress response, particularly that of metal ion stress.

Microbes were the first living cells on Earth. They have co-evolved with the planet and thus have experienced a wide range of geological changes that have produced a wide variety of ecological niches and stressors. Stressors to microbial life include expected physical and chemical concerns such as: temperature, pressure, redox state, pH, ionic strength, osmolarity, UV light exposure and shear forces. Additionally, other stressors exist such as low availability of key nutrients, carbon sources, scarcity in electron acceptors, antimicrobial compounds, and also anthropogenic-produced or released pollutants that include toxic metals.

A wide range of natural extreme environments that are inhabited by extreme microorganisms are present world-wide. However, Chile has remarkable diversity, from the Atacama Desert and Altiplano highlands in the North to Patagonia and Antarctic regions in the South, and from South Pacific and Rapa Nui Island in the West to the Andes Mountains in the East [1]. In addition, polluted environments due to anthropogenic activities (e.g., mining, petroleum transport and processing, chemical industries, agriculture, forestry, and aquaculture) generate extreme conditions and are of increasing concern. As such, Chilean scientists have explored such niches in various fields, particularly microbiology.

Stress from such sources is a curious phenomenon for microbes. Microbes acquired tolerance mechanisms towards various geological epoch stressors, which may or may not be present in the Anthropocene [2]. Certainly, unique physiologies from evolved genomics exist for those species remaining in extreme ecological niches similar to those of the early Earth. Yet new challenging niche environments now exist that have more rapidly been established due to human activities, making this field of study extremely interesting.

Generally, bacteria deal with such stressors by living in a surface attached or agglomeration community referred to as a biofilm. Biofilms have the characteristic phenotype of tolerating a wide range of stressors from the environment [3]. However, bacteria may prefer to migrate from a stressful niche or the presence of toxic molecules or heavy metals [4]. Beyond this, individual strains have evolved specialized genes coding for defined stress resistance determinants. Such determinants are often carried on mobile genetic elements allowing for exchange and optimization of use within a microbial community.

Resistance towards stressors such as temperature, pH, ionic strength and osmolarity are typically from a wide range of physiological adaptations. From presence of unique lipids, transporters, chaperones, and the evolved sequences of the proteins that are exposed to the stress. Systematic environmental microbiology followed up by molecular microbial research has led to advances in understanding such stress responses. These studies have also led to outstanding biotechnological advances such as thermostable DNA polymerases, enzymes for green-chemistry that work at alkali and acidic pH or in extremes of ionic strength, synthesis of special biomolecules (e.g., antioxidants) and biopolymers such as polyhydroxyalkanoates, microorganisms and extremophiles for the clean-up of polluted environments [5] and the protection and alleviation of stress in plants [6].

Organic molecules as stressors come in many forms including, but not limited to: chemical modifications of natural antibiotics, a wide range of chemically synthesized antiseptics, biocides, herbicides and a plethora of pollutants. The diverse genetic diversity of the remarkable number of microbial species on the planet usually means that a species somewhere has evolved the ability to use such compounds as a carbon and/or energy source. Metabolic genes and mechanisms for such activities have been studied since the 1970s when scientists began to realize that microbes are remarkable chemists. Biofilms also provide strategies to survive in polluted environments [7]. This provides us with the field of bioremediation to clean up the sloppiness of the industrial activity. However, xenobiotic pollutants are of concern as they have been modified with atoms and bonds that are not within the natural enzymatic realm. It is thus impressive how well many microbes tolerate such challenges via changes in membrane composition, through efflux pumps, or via biotransformation and biodegradation using evolved metabolic enzymes and pathways [8].

Organisms as diverse as bacteria, yeast, and mammals share a requirement for the regulation of cellular content of essential metals to ensure correct function of several metal-binding proteins, like copper, iron, nickel, and zinc. Recently, molecular approaches have led to the discovery of diverse mechanisms underlying metal homeostasis [9]. We now have an understanding of some of the processes involved in metal uptake, transport, and removal, and are beginning to understand the regulation of these processes [9, 10]. For toxicity we tend to see modes of physiological disturbance including: disrupting essential metal homeostasis, displacing essential metals in enzymes, disruption of cytochromes and/or [Fe–S] centres releasing iron that catalyses Fenton reactions generating reactive oxygen species. Most stressors lead to some amount of ROS production, but metals that can catalyse the reactions lead to high ROS loads that damage DNA, proteins, and membranes. To prevent the consequences of metal overload, living organisms have evolved molecular mechanisms that regulate uptake, intracellular traffic, storage, and efflux, reduction/oxidation to change species, chemical modification, sequestration, repair of damage (typically from ROS) [11–14]. Response to metal stress has seen increased interest in regard to microbiology in mine leachates and tailing ponds, bioleaching in high-value metal mining, to new directions of using non-essential metals as antimicrobials or using microbes to produce metal nanomaterials [15, 16]. Regardless, there is still far more to learn about the mechanisms of toxicity and resistance for specific metals in selected strains, particularly how bacteria survive metal pollutants [17].

From above we see the importance of this field. As such, many microbiologists, whether clinical, environmental, or molecular find themselves studying microbial response and tolerance to stressors at one time or another in their carriers. The late Professor Claudio Christian Vásquez Guzmán was a prominent and talented scientist from Chile. Claudio Vásquez studied biochemistry at the Universidad de Chile (1977) and received his Ph.D in Biological Sciences from the Pontificia Universidad Católica de Chile (1983). He started his academic career as an Assistant Professor at Universidad de Chile (1985–1988), continued as an Associate Professor at Universidad de Talca (1988–2005), and became a Full Professor at Universidad de Santiago de Chile (2005–2020). During his academic career, Claudio and his team published close to 100 internationally recognized papers, and trained an important number of researchers and students including 6 postdoctoral researchers, 28 Ph.D, 9 M.Sc, and 44 undergraduate students. He also influenced and mentored junior scientists from around the world. Several of his former trainees have contributed to this special issue. We acknowledge his disciples and colleagues, and particularly Felipe Arenas, José Manuel Pérez-Donoso and Claudia Saavedra, for their generous support and for providing valuable information and the photo for this special issue.

Claudio was a generous colleague and friend that always provided helpful and creative scientific advice, sharing with generosity his knowledge and experience, and mentoring on how to proceed given diverse scientific challenges.

Michael Seeger: Prof. Claudio Vasquez was my professor in a molecular biology course I took in 1987 as part of my major in biochemistry at Universidad de Chile. It was a difficult time to live in Chile, as it had been controlled by a brutal dictatorship since 1973. Claudio Vasquez, along with his colleague Enrique Gonzalez (Kiko), were a dynamic team of professors that were often smiling and fascinated us. They taught molecular biology, its theory and new experimental techniques, and always encouraged and maintained friendly relationships with undergraduate students. Michael Seeger and Mauricio González: Later, we cooperated with Claudio in diverse national scientific symposia and meetings, as members of local evaluation committees for funding (Fondecyt), and a number of Ph.D theses committees. Raymond J. Turner: My interactions with Prof. Vásquez fall in the area of tellurite resistance and related microbial biochemistry and physiology. We communicated often during the 2000s, soon after I started my position at the University of Calgary, Canada. He was a tough reviewer of my manuscripts for sure, and it was through the personal communications that he helped mentor me to bring my research on tellurite resistance mechanisms to a higher level. Claudio never hesitated to reply to an email of questions and concerns. Through an invitation to contribute a review on tellurium microbiology for a special edition of *Tellurium* in biological systems 10 years after the Fukushima disaster [18], we were able to work together and highlight the discoveries of various enzymes and biochemical processes involved in tellurite-microbe interaction.

The nine articles of this special issue of *Biological Research* address biochemical and genetic determinants of microbial response and tolerance to stressors in different biological models and environmental contexts. Individual articles provide a broad exploration of our current knowledge of response to stressors, with a special emphasis on metal metabolism and toxic compounds. Beginning with genomic approaches, the reader will be provided insights about transcriptional regulation activated in response to stressors by examining the OxyR and SoxR transcriptional regulators and genetic regulation process using OmpX porin in response to hydrogen peroxide stress as a model. Based on the best available scientific evidence, the cellular mechanism of tellurite control is reviewed, including a study on how tellurite reduction contributes to tolerance against tellurite in a bacterial model. Another two studies examine the basic molecular and cellular mechanism of copper homeostasis. The results suggest that tolerance to copper excess requires a combination of metabolic capabilities, such as inorganic polyphosphate and cobalamin metabolism. From biochemical studies, we also see the multiple dimensions and interrelations of metals (such as lithium and chromate) and metalloids to mechanisms of tolerance and antibiotic resistance. Under real-world conditions, studies using single bacterial models need to be complemented with the exploration of microbial response to stressors using metagenomics approaches. In this context, the results presented in this special issue include information on human gut microbiota associated with chronic kidney disease focusing on resistance to metal(loid)s and antibiotics of microorganisms. Also, at the metagenomics level, an interesting study reports the taxonomic and functional characterization of bacterial communities inhabiting Li-rich extreme environments in the Atacama salt flat. Finally, the special issue contains an article that addresses the challenges to finding molecular targets related to pathogeny, virulence, and survival that could be useful in designing new prophylactic or therapeutic strategies against aquatic pathogenic bacteria important to the health and productive aspects of Chilean salmon farming. Although much has been learned, we still have been unable to entirely characterize the full spectrum of response to stressors that are exhibited by the high diversity of members of the bacterial domain.

This special issue highlights the field of microbial stress responses with novel contributions from colleagues and former trainees of Professor Claudio C. Vásquez Guzmán as a tribute to his legacy. The scientific and academic career and the valuable lessons of Professor Claudio C. Vásquez Guzmán illuminate the present and future studies of microbial stress in diverse fields.
